# Cloning and Characterization of the Human Integrin β6 Gene Promoter

**DOI:** 10.1371/journal.pone.0121439

**Published:** 2015-03-27

**Authors:** Mingyan Xu, Xihe Chen, Hao Yin, Liqin Yin, Fan Liu, Yucai Fu, Jiangwu Yao, Xiaoling Deng

**Affiliations:** 1 Department of Oral Biology and Biomaterial, Xiamen Stomatological Research Institute, Xiamen Stomatological Hospital, Fujian, China; 2 Department of Basic Medical Science, Xiamen University Medical College, Xiamen, Fujian, China; 3 Laboratory of Cell Senescence, Shantou University Medical College, Shantou, Guangdong Province, China; University of Massachusetts Medical, UNITED STATES

## Abstract

The integrin β6 (ITGB6) gene, which encodes the limiting subunit of the integrin αvβ6 heterodimer, plays an important role in wound healing and carcinogenesis. The mechanism underlying ITGB6 regulation, including the identification of DNA elements and cognate transcription factors responsible for basic transcription of human ITGB6 gene, remains unknown. This report describes the cloning and characterization of the human ITGB6 promoter. Using 5′-RACE (rapid amplification of cDNA ends) analysis, the transcriptional initiation site was identified. Promoter deletion analysis identified and functionally validated a TATA box located in the region −24 to −18 base pairs upstream of the ITGB6 promoter. The regulatory elements for transcription of the ITGB6 gene were predominantly located −289 to −150 from the ITGB6 promoter and contained putative binding sites for transcription factors such as STAT3 and C/EBPα. Using chromatin immunoprecipitation assays, this study has demonstrated, for the first time, that transcription factors STAT3 and C/EBPα are involved in the positive regulation of ITGB6 transcription in oral squamous cell carcinoma cells. These findings have important implications for unraveling the mechanism of abnormal ITGB6 activation in tissue remodeling and tumorigenesis.

## Introduction

Integrins are heterodimeric trans-membrane proteins that are composed of non-covalently associated *α* and *β* subunits [1]. To date, 18 *α* and 8 *β* subunits have been identified and are capable of forming more than 24 heterodimers that account for the structural and functional diversity of the integrin family [2]. Integrins not only mediate cell-to-cell and cell-to-extracellular matrix (ECM) interactions, they also serve as signaling molecules by mediating outside-in and inside-out signaling [3] that regulate diverse process such as proliferation, differentiation, migration, cell survival, tumor invasion, and metastasis [4,5].

The integrin αvβ6 is restricted to the epithelium and is a receptor for both latency-associated peptide (LAP) of TGF-β and ECM proteins such as fibronectin and vitronectin [6]. Normally, αvβ6 plays an important role in fetal development and wound healing [7]; however, it is also involved in pathological processes such as tumor invasion and tissue fibrosis [2,8,9]. αvβ6 is not constitutively expressed in healthy epithelia, but has been shown to be up-regulated during tissue remodeling, including wound healing and carcinogenesis [4]. Increased expression of αvβ6 has also been detected in carcinomas of the lung, breast, colon, stomach, endometrium, ovary, salivary gland, as well as skin and oral squamous cell carcinoma (OSCC) [8], and is often associated with tumor invasion and metastasis. αvβ6 expression depends on integrin β6 (ITGB6) expression, as ITGB6 only partners with αv forming a single heterodimer. Therefore, it is essential to understand the mechanisms underlying the regulation of ITGB6 expression.

ITGB6 expression is primarily regulated at the level of transcription initiation[10]. Although it has been reported that transcription factors, including Ets-1 [11], signal transducer and activator of transcription 3 (STAT3) [12], Smad3 and AP-1 [13], mediate the initiation of ITGB6 expression under certain clinical conditions, the mechanism and regulatory components that control ITGB6 transcription regulation are unknown. Moreover, little is known about the misregulation of ITGB6 transcription in OSCC cells, given that ITGB6 expression is abnormally high. In this study, the cloning and functional characterization of the human ITGB6 promoter is described. A functional TATA box was identified in ITGB6 promoter upstream of the transcription-initiation site (TSS). Furthermore, the transcription factors CCAAT/enhancer-binding protein α (C/EBPα) and STAT3 were found to be involved in the basic positive transcriptional regulation of ITGB6 in OSCC cells.

## Materials and Methods

### Cell culture

The human embryonic kidney cell line 293T and the human OSCC cell line TCA8113 were purchased from China Center for Type Culture Collection. SAS cells were isolated from a poorly differentiated human squamous cell carcinoma [14] and were kindly provided by Professor Jinhua Zheng of Harbin Medical University, China [15]. Cells were cultured in high-glucose Dulbecco′s modified Eagle′s medium (DMEM) containing 10% fetal bovine serum (FBS), 100 U/ml penicillin, and 0.1 mg/ml streptomycin, at 37°C in a 5% CO_2_ incubator. DMEM, FBS, penicillin, and streptomycin were all purchased from Gibco (Genewindows Biotech, Guangzhou, China).

### 5′-Rapid amplification of cDNA ends (5′-RACE)

5′-RACE was performed with a 5′-Full RACE kit with tobacco acid pyrophosphatase (TAP) according to the manufacturer′s instructions (TaKaRa Bio, Dalian, China). ITGB6 cDNA was obtained by a nested PCR approach using LA Taq DNA polymerase (TaKaRa Bio, Dalian, China). The first round of PCR was performed using the 5′-RACE outer primer (5′-CATGGCTACATGCTGACAGCCTA-3′) and an antisense ITGB6-specific primer (5′-AGGCAGTCTTCACAGGTTTCTGC-3′). The second PCR amplification was carried out using 2 μl of the first-round PCR product as template DNA with the 5′-RACE inner primer (5′-CGCGGATCCACAGCCTACTGATGATCAGTCGATG-3′) and a nested antisense ITGB6-specific primer (5′-GCAAAGCAGTTCAATCCCCATTCG-3′). The PCR products were purified, cloned into the pMD-18T vector (TaKaRa Bio, Dalian, China), and sequenced to identify the TSS.

### Cloning of the human ITGB6 promoter reporter constructs

A 1117-bp DNA fragment, corresponding to the region −909/+208 of ITGB6 (the transcription start site was designated as +1), was amplified from DNA obtained from OSCC cells by PCR using the primers sense −909 and antisense +208 listed in Table 1. A series of 5^′^-deletion fragments (−718/+208, −421/+208, −150/+208, −3/+208, −421/−150, −351/−150, −289/−150, −257/−150, −219/−150, and −189/−150) were then generated by a PCR strategy using the -909/+208 PCR product as template and the primers listed in Table 1. All PCR products were gel purified, digested with *KpnI* and *XhoI* (TaKaRa Bio, Dalian, China), and subcloned into the pGL2-basic firefly luciferase vector (Promega, Guangzhou, China).

**Table 1:** Primers used for ITGB6 promoter reporter constructs.

**Construct**	**Primer**	**Sequence (5' > 3′)**
pGL2-B6(−909/+208)	sense −909	CGGGGTACCCTCCTCAGACATAAGCCTGGCC
pGL2-B6(−718/+208)	sense −718	CGGGGTACCTCACTCTTTCACTCAACAAATA
pGL2-B6(−421/+208)	sense −421	CGGGGTACCTTCCCTAGCCTTCCTTCTCATT
pGL2-B6(−150/+208)	sense −150	CGGGGTACCAGGATGCAGAGAGACTCATAGA
pGL2-B6(−3/+208)	sense −3	CGGGGTACCCTGTCCAGGTAGCCTCTGTT
	antisense +208	CCGCTCGAGCCCATTCGTTTCAGTTCTTGC
pGL2-B6(−421/−150)	sense −421	CGGGGTACCCTCCTCAGACATAAGCCTG
pGL2-B6(−351/−150)	sense −351	CGGGGTACCGCAAAGGATTTAGCAATGAAAC
pGL2-B6(−289/−150)	sense −289	CGGGGTACCTTCCTCATATATAGCCTATATGC
pGL2-B6(−257/−150)	sense −257	CGGGGTACCTCTTTATTCTTTACATTAAATTAAAATTA
pGL2-B6(−219/−150)	sense −219	CGGGGTACCTGACTATATTTCTATTGCTG
pGL2-B6(−189/−150)	sense −189	CGGGGTACCTCATTGATCAATATTTCAC
	antisense −150	CCGCTCGAGGGTGTAAGTTCTATGAGTCTCT

Site-directed mutagenesis was used to inactivate the individual transcription factor-binding sites in the ITGB6 promoter, which was performed by an overlapping PCR-based approach [16]. Primers used were listed in Table 2. All plasmid constructs were confirmed by DNA sequencing.

**Table 2:** Primers used to generate ITGB6 promoter mutations.

**Construct**	**Primer**	**Sequence (5' > 3′)** ^a^
pGL2-B6-M-TA	mut TBP2 F	GAAGTTGCTTTGCAACACAGCTTT
	mut TBP2 R	AAAGCTGTGTTGCAAAGCAACTTC
pGL2-B6-M-STAT3(1)	mut STAT3(1)F	CGGGGTACCTTCCTCATATATAGCCTATATGCTGGCTTTAATCTTTATTC
	antisense-150	CCGCTCGAGGGTGTAAGTTCTATGAGTCTCT
pGL2-B6-M-STAT3(2)	mut STAT3(2)F	CTTTACATTAAATTAAAATTAGGGTTAAAATGACTATATTTCTATTGC
	mut STAT3(2)R	GCAATAGAAATATAGTCATTTTAACCCTAATTTTAATTTAATGTAAAG
pGL2-B6-M-STAT3(3)	mut STAT3(3)F	GATCAATATTTCACTCCAGATATTAACTTTCTCTAGGATGCAGAGAGAC
	mut STAT3(3)R	GTCTCTCTGCATCCTAGAGAAAGTTAATATCTGGAGTGAAATATTGATC
pGL2-B6-M-C/EBPα	mut C/EBP F	ATGACTATATTTCTCGTGCTGTTGTGACTTTTCAT
	mut C/EBP R	ATGAAAAGTCACAACAGCACGAGAAATATAGTCAT

^a^ mutation sites are underlined

### Transient transfection and dual luciferase reporter assay

For promoter activity analysis, 293T and TCA8113 cells (4–5 × 10^4^/well) were seeded in 96-well plates. About 100 ng of each plasmid (combined with 0.25 ng of pRL-TK) was transfected into cells using Metafectene pro transfection reagent (Biontex Laboratories GmbH, Germany), according to the manufacturer′s protocol. Cells were harvested after 48 hr. Firefly and Renilla luciferase activities were determined using a dual-luciferase reporter assay system (Promega, Guangzhou, China) and analyzed using a Lumat LB9507 luminometer (Berthold, Wildbad, Germany). Firefly luciferase activity was normalized to Renilla luciferase readings in each well. Each experiment was conducted at least twice in triplicate. For co-transfection, cells (2–3 × 10^4^/well) were seeded in 96-well plates and transfected with 5 pmol siRNA by Metafectene siRNA transfection reagent (Biontex Laboratories GmbH, Germany) according to the manufacturer′s protocol. After 24 hr, 100 ng of each plasmid (combined with 0.25 ng of pRL-TK) was transfected into cells using Metafectene pro. Cells were harvested after 48 hr.

For western blot and mRNA isolation, cells were seeded in 6-well plates (3–4 × 10^5^/well) and transfected with 150 pmol siRNA using Metafectene siRNA transfection reagentaccording to the manufacturer′s protocol. Cells were harvested after 48 hr.

### Electrophoretic mobility shift assays (EMSA)

Nuclear extracts from TCA8113 and 293T cell were prepared as previously described [17]. For each preparation, 5 × 10^6^ cells were used. All procedures were carried out at 4°C. The single-strand 5′-biotin end-labeled oligonucleotides, unlabeled oligonucleotides and labeled mutant oligonucleotides were synthesized by TaKaRa Bio Company and annealed. EMSA was carried out with the Light Shift Chemiluminescent EMSA Kit (Thermo Fisher scientific, Guangzhou, China) according to manufacturer’s instructions. The binding reaction consisted of 10 fmol labeled double-stranded oligonucleotide (wild type or mutated) and 1 μl of nuclear extract in a total volume of 20 μl. The whole reaction was run on a 5% polyacrylamide gel and electrophoretically transferred to a nylon membrane (Thermo Fisher scientific). The transferred DNA was cross-linked to the membrane by using a UV light for 30 min, detected by chemiluminescence and exposed to X-ray film (Kodak, Shanghai, China) for 2 min. For a specific competitor, 2 pmol unlabeled wild-type oligonucleotide (100-fold excess) was used.

### Chromatin immunoprecipitation (ChIP) assay

In order to analyze the *in vivo* association of transcriptional factor with the ITGB6 gene promoter, ChIP was performed as previously described [18]. About 1 × 10^7^ TCA8113 cells were fixed using 1% formaldehyde for 10 min at room temperature (RT) to cross-link proteins to DNA, after which 125 mM glycine solution was added for 5 min at RT to quench the formaldehyde. The fixed cells were washed, lysed in lysis buffer containing protease inhibitor cocktail (Sigma, Shanghai, China), and sonicated (Sonifier Cell disruptor 350, Branson Sonic Power Co, MA) at 30% of maximum power, 3 times for 15 sec each, with a 30-sec cooling interval. The supernatant containing chromatin was collected and further diluted 10-fold in the ChIP dilution buffer [0.01%, SDS, 1.1% Triton X-100, 1.2 mM EDTA, 16.7 mM Tris–HCl (pH 8.1), 167 mM NaCl, and protease inhibitor cocktail]. About 10 μl of the diluted DNA fraction was kept aside as template for PCR. The remaining DNA fraction was precleared using 25 μl of protein G magnetic beads for 2 hr at 4°C. Immunoprecipitation was performed by adding 2 μg of the antibody (anti-TBP [GTX60341, GeneTex, Neobioscience, Shenzhen, China]; anti-C/EBPα [sc-61, Santa Cruz Biotechnology, Inc., Univ-bio, Guangzhou]; anti-STAT3 [sc-482, Santa Cruz Biotechnology, Inc.]; normal rabbit IgG [sc-66931, Santa Cruz Biotechnology, Inc.] as a negative control). After appropriate washing, the antibody-transcription factor-DNA complex was eluted from the beads, formaldehyde cross-links were reversed, and proteins were digested with proteinase K at 67°C for 2 hr. DNA was purified and used for PCR using the following primers for TBP (F 5′-GCAGAGAGACTCATAGAACTT-3′; R 5′-AACAGAGGCTACCTGGAC-3′) and C/EBPα and STAT3 (F 5′-TTCCTCATATATAGCCTATATGC-3′; R 5′-GGTGTAAGTTCTATGAGTCTCT- 3′) experiments.

### Western blot analysis

Proteins were isolated with RIPA buffer (0.05 M Tris-HCl, 150 mM NaCl, 1% NP-40, 1% sodium deoxycholate, 0.1% SDS) containing a cocktail of protease inhibitors (Sigma-Aldrich), separated by sodium dodecyl sulfate-polyacrylamide gel electrophoresis (SDS-PAGE) and transferred to a nitrocellulose membrane. The membrane was blocked by non-fat milk followed by incubation with anti-TBP (GeneTex), anti-β-actin (sc-8423, Santa Cruz Biotechnology, Inc.), anti-GAPDH (Goodhere, Hangzhou, China), anti-STAT3, or anti-C/EBPα antibody at 4°C overnight. After incubating with a peroxidase-conjugated goat anti-rabbit or anti-mouse IgG, protein bands were visualized by Western Bright ECL (Advansta, California, USA).

### RNA isolation and RT-qPCR

Total RNA was isolated from cells by using a General RNA Extraction Kit (Dongsheng, Biotech, Guangzhou, China), according to the manufacturer’s protocol. Total RNAs were reverse transcribed using a Fast Quant RT Kit (Tiangen Biotech, Beijing, China) and RT-qPCR reactions were performed by using a SuperReal PreMix (Tiangen Biotech) on ABI StepOne system (Applied Biosystems, California, USA). The relative mRNA expression was calculated using the 2^-ΔΔCt^ method[19]. Ct data were normalized to the internal standard β-2m. Primers used for RT-qPCR are listed in Table 3.

**Table 3:** Sequences of oligonucleotide primers used in RT-qPCR.

**Gene**	**Forward primer (5' > 3')**	**Reverse primer (5' > 3')**
β-2M	AATCCAAATGCGGCATCT	GAGTATGCCTGCCGTGTG
TBP	GGCACCACTCCACTGTATCC	CCCAGAACTCTCCGAAGCTG
STAT3	GGTCTGGCTGGACAATATCATT	GAGGCTTAGTGCTCAAGATGG
C/EBPα	GGTGGACAAGAACAGCAACGA	GCGGTCATTGTCACTGGTCAG
ITGB6	GCAAGCTGCTGTGTGTAAGGAA	CTTGGGTTACAGCGAAGATCAA

### Statistical analysis

The data are presented as the mean ± standard error of the mean (SEM). Statistical differences between sample means were analyzed using unpaired, two-tailed Student’s t test for single-group and one-way ANOVA for multiple-group comparisons. All statistical analyses were performed using GraphPad Prism 5 software, where *p-*values less than 0.05 were considered statistically significant.

## Results

### Identification of the ITGB6 transcription start site (TSS)

**Fig 1:**
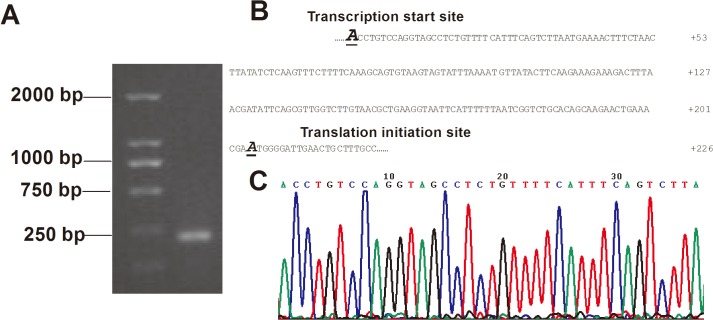
Identification of the transcriptional start site of the human ITGB6 gene. (A) Gel analysis of 5′-RACE experiments on total RNA from oral squamous cell carcinoma cells. Lane 1: DL2000 Marker, Lane 2: a single amplicon detected by 2% agarose gel electrophoresis. (B) The portion of the human ITGB6 nucleotide sequence depicting the positions of the translation initiation site and transcription start site. (C) Sequencing result of 5′-RACE.

As the precise position of the mRNA TSS is critical for the characterization of a promoter region [20], 5′-RACE was performed to amplify the 5′-end of the ITGB6 cDNA from mRNA extracted from OSCC cells. After reverse transcription and nested PCR, a single band of approximately 180-bp was obtained (Fig. 1A), which was then gel-purified and cloned into the pMD18-T vector. Sequencing results from 10 randomly selected clones revealed that a single TSS was located 204-bp upstream of the ATG translation-initiation site in the ITGB6 cDNA (Fig. 1 B and C).

### Cloning and deletion analysis of the ITGB6 promoter

**Fig 2:**
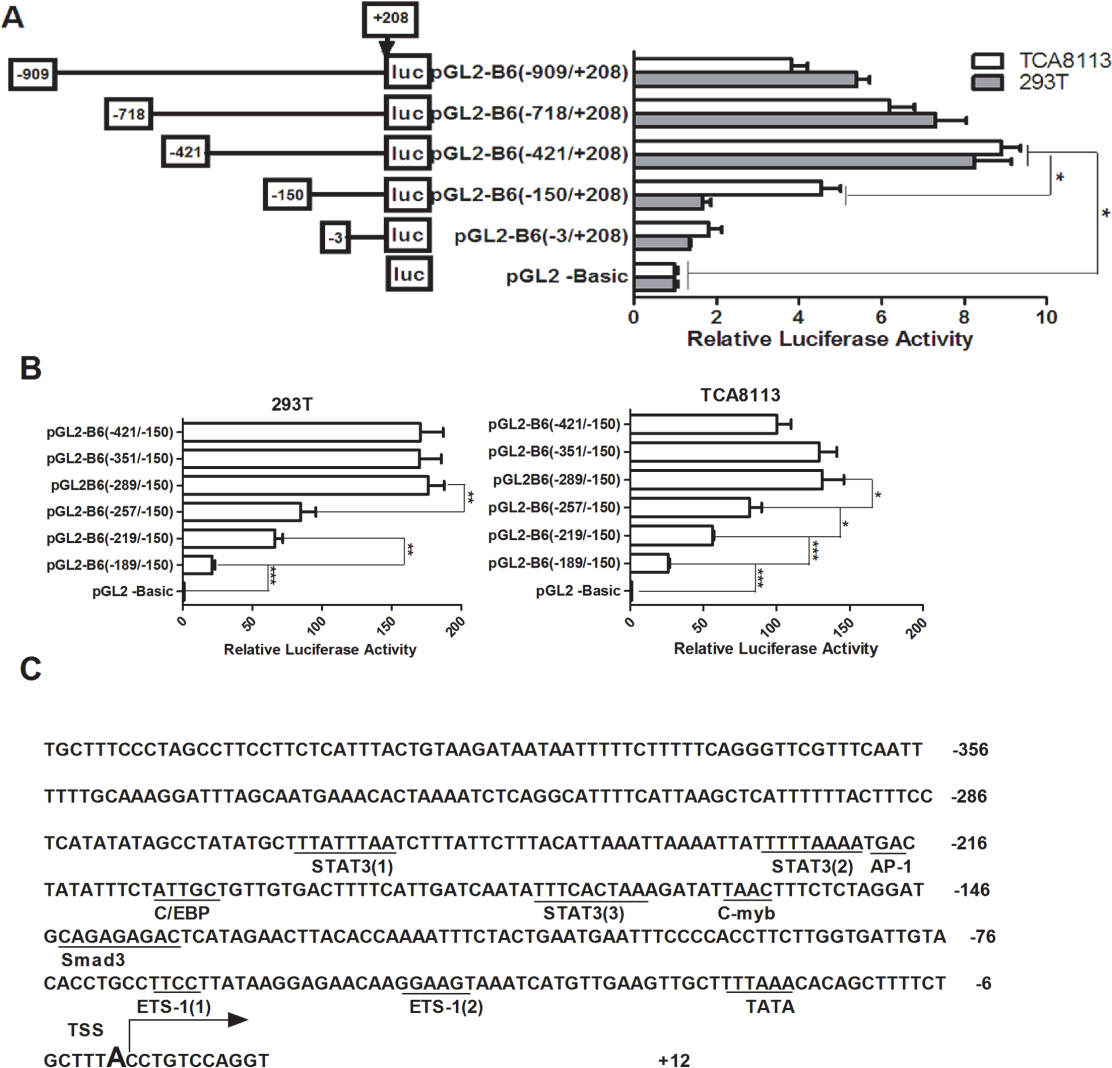
Luciferase reporter assay for the human ITGB6 gene promoter. (A&B) The ITGB6 promoter-luciferase constructs are schematized. All numbers presented are relative to the TSS of the human ITGB6 gene. Luciferase reporter constructs containing various lengths of the ITGB6 promoter, or the promoter-less plasmid pGL2-basic, were co-transfected with pRL-TK into TCA8113 cells and 293T cells. Luciferase activity was measured at 48 hr after transfection. The luciferase value of the pGL2-basic was set to a value of one. Relative promoter activities are presented on the right (mean ± SEM, at least three independent experiments) and are analyzed by one-way ANOVA, where * *p* < 0.05, ** *p* < 0.01, and ** *p* < 0.001. (C) Nucleotide sequence of the main promoter region of the ITGB6 gene, depicting predicted binding sites for the transcriptional factors (underlined).

To investigate the sequence composition of the 5′-flanking region on the promoter activity of ITGB6, a series of 5′-delection fragments of the ITGB6 promoter were PCR-amplified and subcloned into the promoter-less luciferase reporter vector, pGL2-Basic. These constructs were then transfected into a non-human squamous cell carcinoma cell line (293T) and human OSCC cell line (TCA8113) to determine the contribution of each region of the ITGB6 promoter on gene expression activity. A similar level of expression was observed using the −909 and −421 regions in both cell lines. However, when the region between −909 and −421 was deleted, the −421/+208 region of ITGB6 displayed maximum luciferase activity (Fig. 2A). A deletion from −421 to −150 resulted in 50–80% reduction in luciferase activity, whereas further deletion from −150 to −3 nearly abolished the promoter activity. These data suggest that the region between −421 and −150 is the main sequence that positively regulates ITGB6 transcription, whereas the region between −150 and −3 is necessary and required for basal transcriptional activity of human ITGB6 gene.

To further delineate the regulatory elements that positively regulate ITGB6 transcription, a series of 5′-deletions of the −421/−150 fragment were cloned (pGL2-B6[−421/−150]; pGL2-B6[−351/−150]; pGL2-B6[−289/−150]; pGL2-B6[−257/+150]; pGL2-B6[−219/−150]; pGL2-B6[−189/−150]) and analyzed for luciferase expression. Truncation of 5′-flanking sequences from −289 to −150 resulted in a significant decrease in luciferase expression by 74.8% and 68.2% in the 293T in TCA8113 cell lines, respectively (Fig. 2B). These results indicate that the regulatory elements for human ITGB6 positive transcription are located within the −289 to −150 region of the human ITGB6 gene.

To identify regulatory elements involved in basal transcriptional activity and positive regulation of ITGB6, sequence analysis were performed by TRANSFAC-TESS and AliBaba2 analysis software. A TATA box element TTTTAAA was identified between −24 and −18 bp upstream of the TSS (Fig. 2C). Several putative transcription factor-binding sites were also predicted in the −289 to −150 region, including C/EBPα, STAT3, AP-1, and c-Myb (Fig. 2C). Experimental validation of both c-Myb- and AP1-binding sites by mutation analysis did not show any significant effect on promoter activity when compared with the wild type (S1 Fig.).

### Involvement of a functional TATA box in the transcriptional regulation of ITGB6

**Fig 3:**
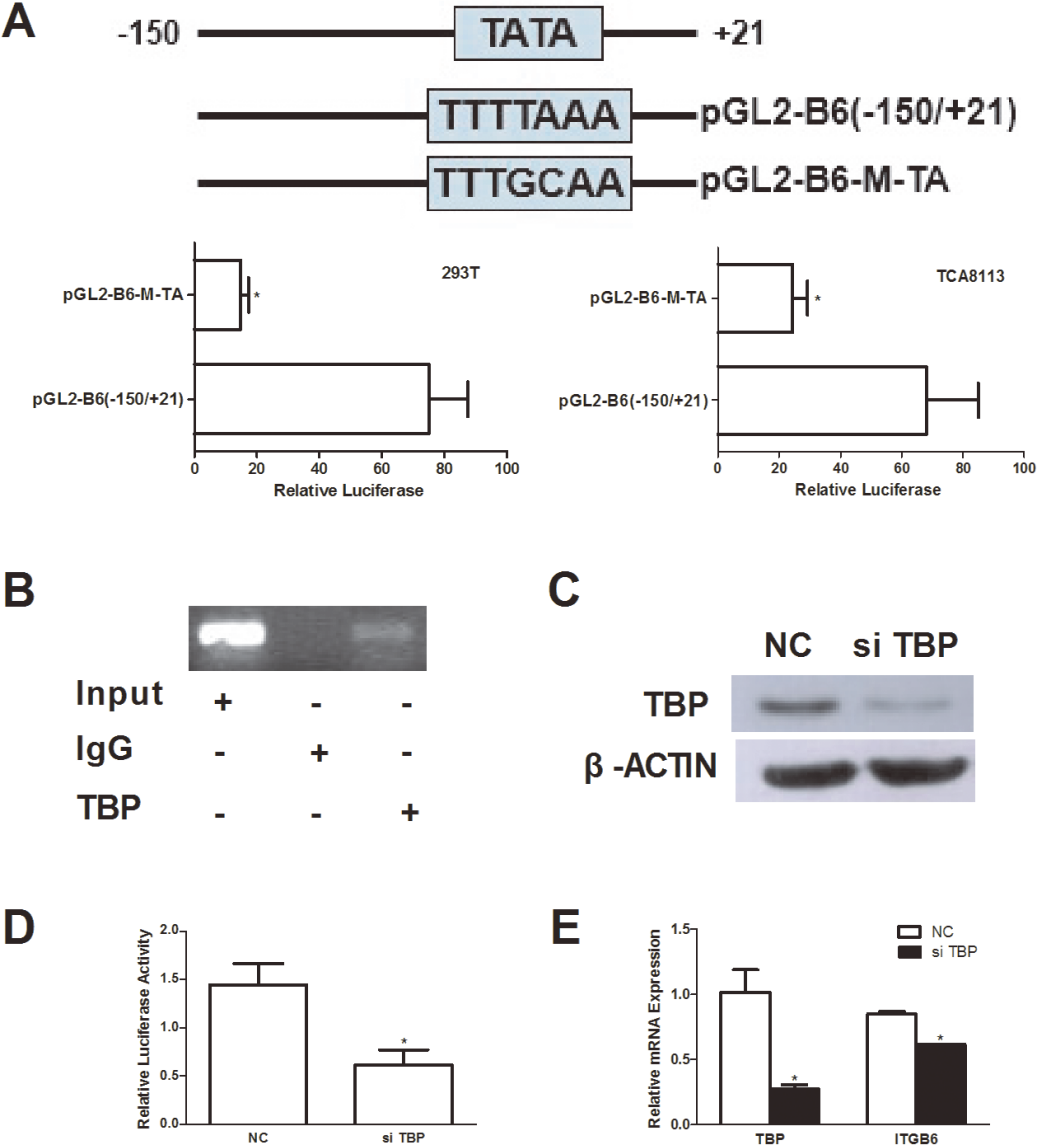
Involvement of TBP in the transcriptional regulation of ITGB6. (A) Luciferase activity expressed by the TATA box directed mutant pGL2-B6-M-TA and wild type construct pGL2-B6(−150/+21) after being transfected into TCA8113 and 293T cells 48 hr. * *p* < 0.05 *vs*. WT. (B) ChIP assay was performed using anti-TBP antibody or IgG as a control in TCA8113 cells. Input and immunoprecipitated DNAs were then amplified by PCR using primer pairs covering the TATA box from −150 to +21. (C) TCA8113 cells transfected with siRNA against TBP, and the TBP protein expression determined by immunoblot analysis. (D) TCA8113 cells were co-transfected with pGL2-B6(−150/+21) and TBP siRNA or non-targeting control (NC) siRNA, and with pRL-RK. Relative luciferase activity was detected by dual luciferase reporter assay system. * *p* < 0.05 *vs*. NC siRNA. (E) SAS cells were transfected with TBP siRNA or NC siRNA for 48 hr. The mRNA expression levels of TBP and ITGB6 were determined by RT-qPCR. * *p* < 0.05 *vs*. NC siRNA.

Site-directed mutagenesis analysis of the predicted TATA box-binding site was performed to investigate its role on the basal transcription activity of ITGB6. Transient transfection of a TATA box mutant (pGL2-B6[−150/+21]) resulted in a significant reduction in the promoter activity when compared with the wild type construct (pGL2-B6[−150/+21]) in both 293T and TCA8113 cells (Fig. 3A).

TATA-binding protein (TBP) is the main DNA-binding protein that recognizes and binds a TATA box in TATA-containing gene promoters[21],. To determine if cellular TBP binds to the TATA box in the ITGB6 promoter, a ChIP assay was performed and assayed using a PCR targeting the −150 to +21 region of the ITGB6 promoter. ChIP with an antibody against TBP resulted in a marked enrichment of the ITGB6 promoter DNA, compared to the control IgG (Fig. 3B). To further investigate the role of TBP on ITGB6 promoter activity, TBP was knocked down by siRNA (sense sequence: 5′-UUGAAUAGUGAGACGAGUUTT-3′) in TCA8113 cells (Fig. 3C). As shown in Fig. 3D, the relative luciferase activity of pGL2-B6(−150/+21) was significantly decreased after TBP was knocked down by transfecting siRNA into TCA8113 cells. These results suggest that TBP could bind on TATA box located at −150 to +21 region of human ITGB6 promoter *in vivo* and is required for human ITGB6 promoter transcriptional activity.

To further investigate the role of TBP in the regulation of human ITGB6 mRNA expression, we knocked down TBP in SAS cells that express high levels of ITGB6 mRNA. RT-qPCR analysis demonstrated that the relative mRNA of TBP and ITGB6 was reduced by 73.3% and 27.3%, respectively (Fig. 3E).

### Involvement of STAT3 in the transcriptional regulation of ITGB6

**Fig 4:**
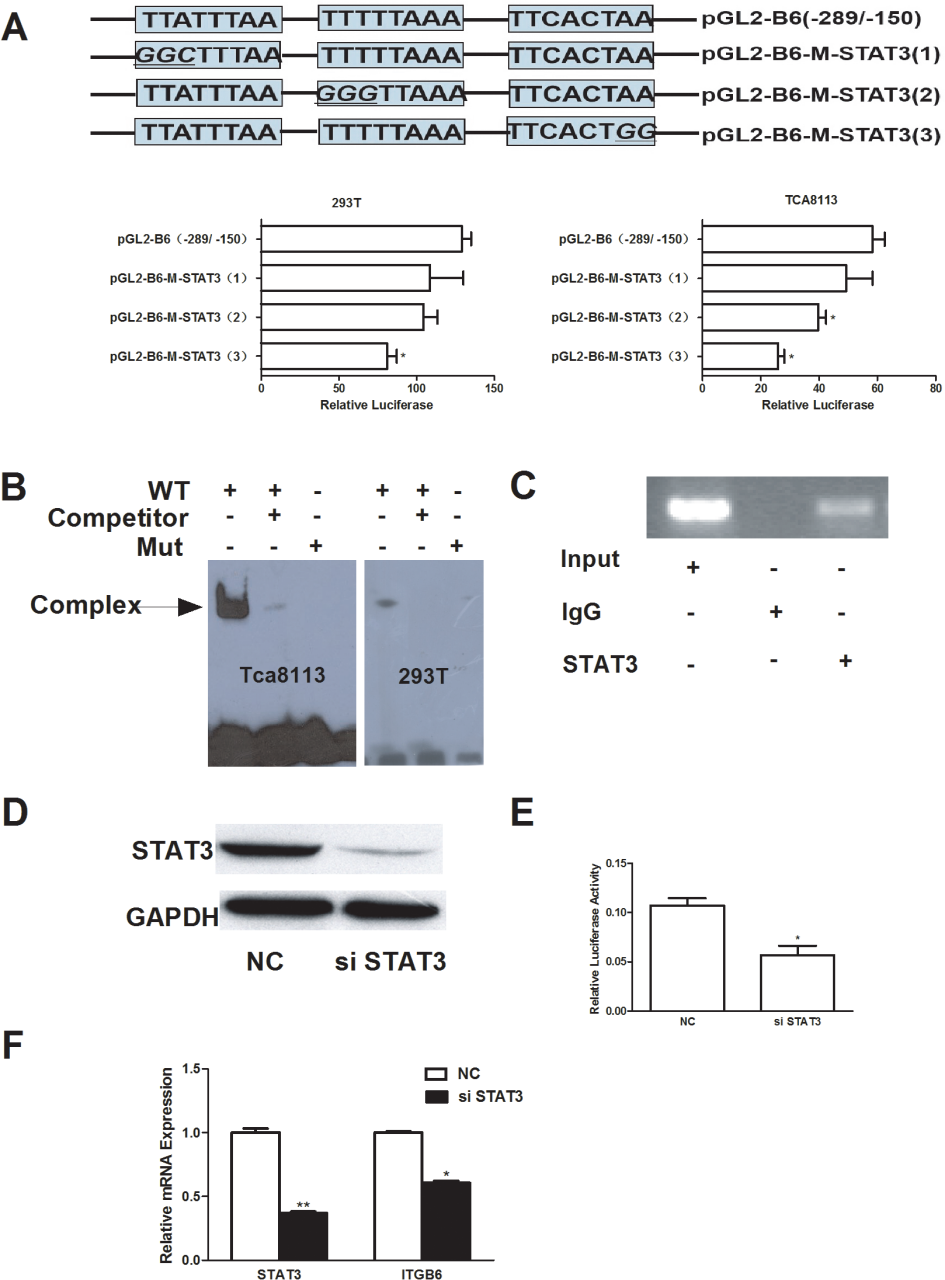
Involvement of STAT3 in the transcriptional regulation of ITGB6. (A) Luciferase activity expressed by TCA8113 and 293T cells after transfection with the potential STAT3-binding site-directed mutants pGL2-B6-M-STAT3(1), pGL2-B6-M-STAT3(2), pGL2-B6-M-STAT3(3) and WT construct pGL2-B6(-289/-150) for 48 hr. * *p* < 0.05 *vs*. WT. (B) EMSA was performed to assay the nuclear extract protein from TCA8113 and 293T cells that binds oligonucleotides containing the third potential STAT3-binding site. (C) ChIP assay was performed using anti-STAT3 antibody or IgG as a control in TCA8113 cells. Input and immunoprecipitated DNAs were amplified by PCR using primer pairs covering the potential STAT3-binding sites from −289 to −150. (D) TCA8113 cells were transfected with NC or STAT3 siRNA for 48 hr, and STAT3 protein production was detected by immunoblot analysis. * *p* < 0.05 *vs*. NC siRNA. (E) Relative luciferase activity was detected after NC or STAT3 siRNA and dual luciferase reporter plasmids were co-transfected into TCA8113 cells for 48 hr. (F) SAS cells were transfected with NC or STAT3 siRNA for 48 hr. STAT3 and ITGB6 mRNA expression were detected by RT-qPCR. * *p* < 0.05, *** *p* < 0.001 *vs*. NC siRNA.

To investigate the role of potential STAT3-binding sites on ITGB6 basal transcription activity, 3 potential STAT3-binding site mutants were made in the parental vector pGL2-B6(−289/−150), namely, pGL2-B6-M-STAT3(1), pGL2-B6-M-STAT3(2), and pGL2-B6-M-STAT3(3) (Fig. 4A). After transient transfection, the relative luciferase activities of these constructs were assayed. The expression of promoter activity was significantly reduced in the pGL2-B6-M-STAT3(3)-transfected cells compared with the control non-mutated construct pGL2-B6(−289/−150) in both TCA8113 and 293T cell lines; however, there was no significant effect on expression when using the pGL2-B6-M-STAT3(1) and pGL2-B6-M-STAT3(2) mutant constructs. These results showed that the third potential STAT3-binding site was the most important for ITGB6 basal transcription activity among these sites. We then performed EMSA to identify whether nuclear transcriptional factors could bind on the third potential STAT3-binding sites. The results showed that the WT 5′-biotin end-labeled oligonucleotides (Sense 5′-bio-CAATATTTCACTAAAGAT-3′, Antisense 5′-bio-ATCTTTAGTGAAATATTG-3′) formed a complex with nuclear extract protein of TCA8113 and 293T cells, whereas non-labeled oligonucleotides and 100× molar excess inhibited complex formation significantly. Moreover, 5′-biotin end-labeled mutational oligonucleotides (Sense 5′-bio-CAATATTTCACT*CCC*GAT-3′, Antisense 5′-bio-ATC*GGG*AGTGAAATATTG-3′) did not form a complex with nuclear extract protein (Fig. 4B). These results further support the role of the third STAT3-binding site as a mediator of ITGB6 transcriptional activity, and show that it is required for transcriptional factor binding *in vitro*.

To further assess whether potential STAT3 DNA-binding motifs of ITGB6 revealed by *in vitro* analysis were functional in the native chromatin, ChIP was performed on chromatin extracted from TCA8113 cells by using anti-STAT3 antibody. PCR targeting the −289 to −150 bp region showed that STAT3 immunoprecipitates were enriched for the ITGB6 promoter DNA compared to the non-specific IgG (Fig. 4C). The role of STAT3 was further validated by siRNA knockdown (sense sequence 5′-GGAGCAGCACCUUCAGGAUTT-3′), which was co-transfected with dual luciferase reporter plasmid pGL2-B6 (−289/−150) and pRL-TK in TCA8113 cells (Fig. 4D). As shown in Fig. 4E, the relative activity of pGL2-B6(−289/−150) was significantly decreased. These results suggest that STAT3 could bind on −289 to −150 region of human ITGB6 promoter *in vivo* and is required for ITGB6 promoter transcriptional activity.

To further determine the role of STAT3 on the regulation of human ITGB6 mRNA expression, STAT3 expression was knocked down by siRNA in SAS cells. The RT-qPCR result showed that the relative mRNA of STAT3 was reduced by 63.8% and the ITGB6 mRNA was reduced by 40.5% (Fig. 4F). These results indicate that STAT3 is required for human ITGB6 mRNA expression.

### Involvement of C/EBPα in the transcriptional regulation of ITGB6

**Fig 5:**
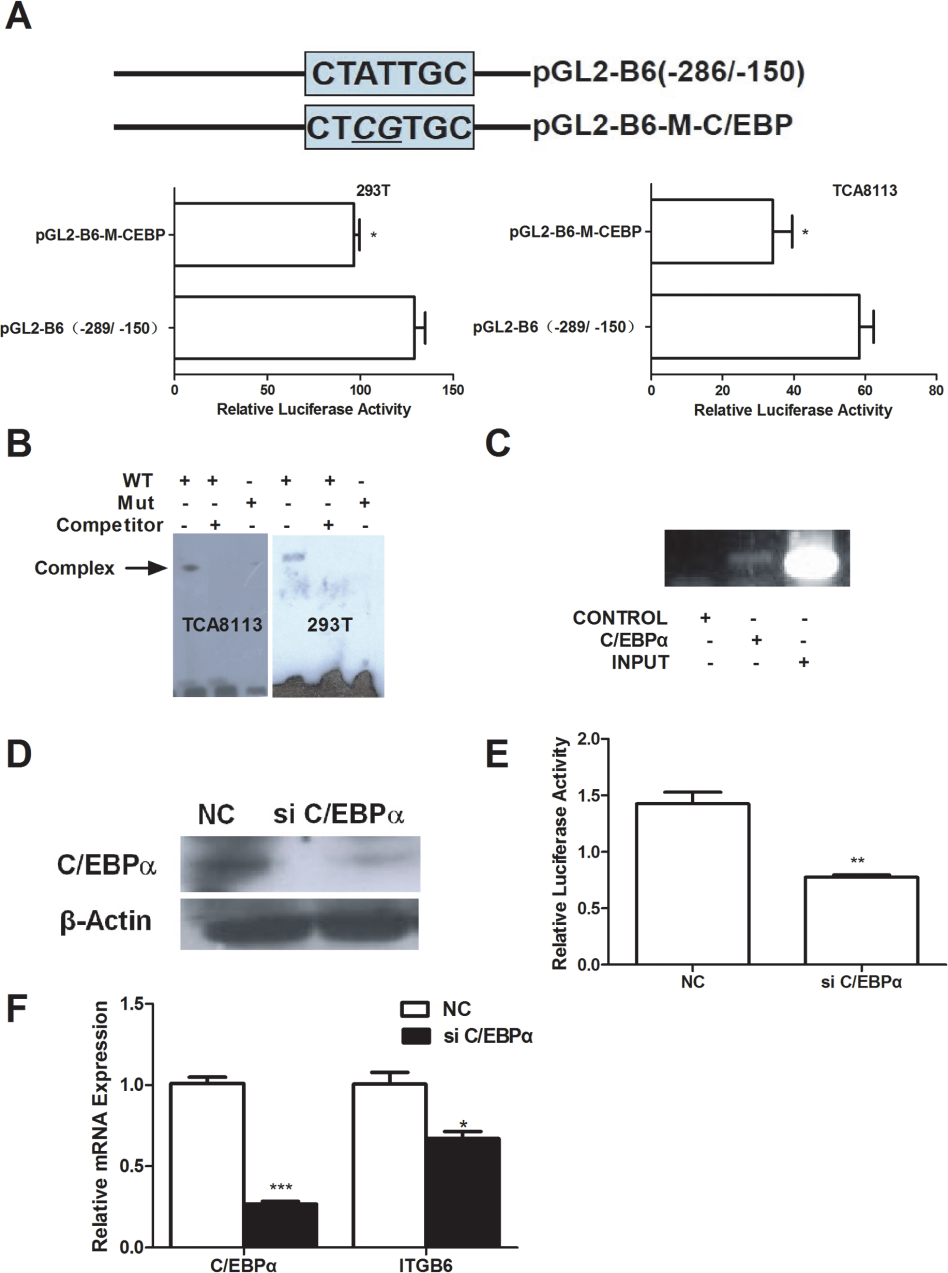
Involvement of C/EBPα in the transcriptional regulation of ITGB6. (A) Luciferase activity expressed by TCA8113 and 293T cells after transfection with the potential C/EBPα-binding site-directed mutants pGL2-B6-M-C/EBPα and the WT construct pGL2-B6(−289/−150) for 48 hr. * *p* < 0.05 *vs*. WT. (B) EMSA was performed to assay TCA8113 and 293T cells′ nuclear extract protein that binding on oligonucleotides containing the C/EBPα-binding site. (C) ChIP assay was performed using anti-C/EBPα antibody or IgG as a control in TCA8113 cells. Input and immunoprecipitated DNA were amplified by PCR using primer pairs covering the C/EBPα-binding site from −289 to −150. (D) TCA8113 cells were transfected with NC or C/EBPα siRNA for 48 hr, and C/EBPα protein was detected by immunoblot analysis. (E) Relative luciferase activity was detected after NC or C/EBPα siRNA and dual luciferase reporter plasmids were transfected into TCA8113 cells for 48 hr. ** *p* < 0.01 *vs*. NC siRNA. (E) SAS cells were transfected with NC or C/EBPα siRNA for 48 hr. C/EBPα and ITGB6 mRNA expression were detected by RT-qPCR. * *p* < 0.05, *** *p* < 0.001 *vs*. NC siRNA.

To investigate the role of potential C/EBPα-binding sites on ITGB6 basal transcription activity, a binding site mutant pGL2-B6-M-C/EBPα in the parental vector pGL2-B6(−289/−150) was generated (Fig. 5A). After transient transfection, the relative luciferase activity of pGL2-B6-M-C/EBPα was significantly reduced when compared with the control non-mutated construct pGL2-B6(−289/−150) in both TCA8113 and 293T cells. EMSA was performed to determine whether nuclear transcriptional factors can bind to the C/EBPα-binding sites. The results showed that the WT 5′-biotin end-labeled oligonucleotides (Sense: 5′-bio-ATTTCTATTGCTGTTGTG-3′; Antisense: 5′-bio-CACAACAGCAATAGAAAT-3′) formed a complex with nuclear extract protein of TCA8113 and 293T cells, whereas 100× molar excess of non-labeled oligonucleotides prevented complex formation. Moreover, 5′-biotin end-labeled mutational oligonucleotides (Sense: 5′-bio-ATTTCT*CGG*GCTGTTGTG-3′; Antisense: 5′-bio-CACAACAGC*CCG*AGAAAT-3′) did not form a complex with nuclear extract protein (Fig. 5B). These results suggested that the putative C/EBPα-binding sites are important for human ITGB6 promoter transcriptional activity and is required for transcriptional factor binding *in vitro*.

C/EBPα has been previously reported to be associated with integrin CD11c promoter activation [22], and therefore, a ChIP assay was performed to determine if C/EBPα still can bind to the ITGB6 promoter in TCA8113 cells. Immunoprecipitates obtained using an antibody against C/EBPα resulted in a marked enrichment of the ITGB6 promoter DNA compared to the non-specific IgG (Fig. 5C). To further investigate the role of C/EBPα in transcriptional regulation of ITGB6 promoter activity, we successfully knocked down C/EBPα using specific siRNA (sense sequence: 5′-GTCGGCCAGGAACTCGTCGTT-3′) that was co-transfected with dual luciferase reporter plasmid pGL2-B6 (−289/−150) and pRL-TK in TCA8113 cells (Fig. 5D). After TCA8113 was transfected with C/EBPα siRNA, the relative luciferase activity of pGL2-B6 (−289/−150) was significantly decreased (Fig. 5E). These results suggest that C/EBPα could bind on the −289 to −150 region of human ITGB6 promoter *in vivo* and C/EBPα is required for human ITGB6 promoter transcriptional activity.

To further determine the role of C/EBPα on the regulation of ITGB6 mRNA expression, the effect of C/EBPα knockdown on ITGB6 mRNA level in SAS cells was investigated. The RT-qPCR result showed that the mRNA expression of C/EBPα was reduced by 74.4% and ITGB6 mRNA expression was reduced by 32.9% (Fig. 5F). These results indicate that C/EBPα is also required for human ITGB6 mRNA expression.

## Discussion

ITGB6 is a critical subunit of the αvβ6 integrin heterodimer and is involved in regulating the expression of the heterodimer during cancer and wound healing. To understand the transcriptional regulation of the ITGB6 gene in cancer cells, we identified and characterized the human ITGB6 promoter and its activity. Our results revealed, for the first time, the presence of functional TATA box located from −24 to −18 bp upstream of the ITGB6 promoter. Furthermore, this study has identified that the transcription factors STAT3 and C/EBPα are both involved in the positive regulation of ITGB6 basic transcription in OSCC cells.

Identification of the TSS has been previously described as the first step in defining the promoter and studying its regulatory mechanisms [23]. Using 5′-RACE, we identified that a single TSS of the ITGB6 gene is located 37-bp upstream of the previously reported position [11,12]. This indicates that the length of the 5′-untranslated region (UTR) of ITGB6 extends at least 37 bp; this increased length suggests that the regulation of ITGB6 mRNA is more complex than previously described, and likely contains additional regulatory elements required to form a larger preinitiation complex when translation begins [24]. The identification of only a single TSS suggests that it is likely to be the major transcriptional start site of ITGB6.

To begin to functionally characterize the sequence upstream of the TSS, a series of 5′-deletions of the ITGB6 promoter were generated. We found that the region between −150 and −3 is minimally required for basal promoter activity. Bioinformatic analyses identified a potential TATA box located −24 to −18 upstream of the TSS. As the core promoter element, the TATA box is the binding site of either general transcription factors or histones, and is involved in transcription by RNA polymerase [25]. Our results showed that the endogenous TBP binds to the region containing the TATA box sequence, suggesting that the interaction of TBP with the TATA box might be required for ITGB6 expression. However, the enrichment of TBP-DNA was not found to be strong; as the binding affinity of TBP to the sequence TTTAAA (the TATA box sequence of human ITGB6 gene) has been shown to be less than the consensus sequence TATAAA [26], the poor enrichment may reflect a lower binding affinity of the TBP-DNA immunoprecipitation due to variation of TATA box sequence. Nonetheless, both mutation of the TATA box and knockdown of TBP expression resulted in a significant reduction of ITGB6 promoter activity. These results support the role of a functional TATA box in ITGB6 transcription, and that the ITGB6 promoter is a TATA promoter. The reduction of luciferase activity in cells containing the promoter deletion constructs pGL2-B6(−150/+208) and pGL2-B6(−3/+208) could be at least in part due to the lack of TATA box.

A region further upstream of the TSS, −289 and −150 bp, was identified as the minimal sequence that holds the most influence over promoter activity. A number of putative binding sites for transcription factors were identified in this region, including STAT3, C/EBPα, AP-1 and c-Myb, however, empirical data from the mutation analysis of the binding sites suggests that only STAT3 and C/EBPα function as the positive regulators of ITGB6 basal transcriptional activity (Figs. 4A, 5A, and S1 Fig.). These results provided rationale to further investigate how the basal transcriptional activity of human ITGB6 gene is regulated by the transcription factors STAT3 and C/EBPα in OSCC cells.

The transcription factor STAT3 is not only persistently activated in many types of cancer, including oral cancer [27,28], but also functions as a transcription factor required for regulating genes that are involved in tumor proliferation, survival, angiogenesis, and invasion. The data from our mutation analysis, EMSA, ChIP assay, and siRNA knockdown experiment indicate that STAT3 is involved in basal transcriptional activation of ITGB6 gene in OSCC cells. The identification and functional characterization of STAT3 in our study is consistent as the previous report that demonstrated that STAT3 was associated with ITGB6 transcriptional activation in both prostate and breast epithelial cells [12]. These data suggest that STAT3 may be an organ-independent transcription factor involved in basal transcriptional activation of ITGB6 in cells of epithelial origin.

The transcription factor C/EBPα belongs to the CCAAT enhancer-binding protein (C/EBPs) family of basic leucine zipper transcription factors that recognize the CCAAT motif of their target gene promoter [29,30]. As the role of C/EBPα was reported to act as tumor suppressor in several tumor types, including head and neck squamous cell cancers [31,32], we initially hypothesized that C/EBPα might repress ITGB6 transcription in OSCC cells. Conversely, our results demonstrate that C/EBPα acts as a positive regulator of ITGB6 gene basic transcription in OSCC cells. Although C/EBPα has been reported to act as a transcriptional activator of other integrin gene promoters such as CD11c [22], C/EBPα-mediated transcriptional activation depends on co-transcription factors such as Sp1 and AP1 [22,33]. However, we did not identify any co-operating factors associated with C/EBPα in this study; this remains to be investigated in future studies.

Our findings do not preclude other transcription factor-binding sites that are located beyond the −289/−150 region characterized that may also be involved in ITGB6 regulation. Indeed, putative binding sequences for transcription factors such as Ets-1 and Smad were identified within the region between −146 and +1, which have been shown to robustly induce ITGB6 promoter activity [11,13]. However, the data presented clearly defines a central role for the TATA box, and STAT3 and C/EBPα transcription factors in the activation of ITGB6 expression in OSCC cells.

In conclusion, we have characterized the promoter region of the human ITGB6 gene. Our results demonstrated, for the first time, that ITGB6 promoter contains a functional TATA box and that the transcriptional factors STAT3 and C/EBPα are involved in the positive regulation of ITGB6 transcription. These findings offer new insights toward understanding the temporal and disease-specific regulation of the human ITGB6 gene in OSCC cells.

## Supporting Information

S1 Fig Mutation analysis of AP1- and c-Myb-binding sites in the −289/−150 region of the ITGB6 promoter. Luciferase activity expressed by TCA8113 and 293T cells after transfection with the potential AP1-binding site-directed mutant pGL2-B6-M-AP-1(A), the potential c-Myb-binding site-directed mutant pGL2-B6-M-cMyb(B), and wild-type construct pGL2-B6(−289/−150) 48 hr post transfection.
(DOC)

S1 File Minimal data set of all figures. (RAR)

## References

[1] Hynes RO (2002) Integrins: bidirectional, allosteric signaling machines. Cell 110: 673–687. 12297042 10.1016/s0092-8674(02)00971-6

[2] Ganguly KK, Pal S, Moulik S, Chatterjee A (2013) Integrins and metastasis. Cell Adh Migr 7. 10.4161/cam.23840 PMC3711990 23563505

[3] Morse EM, Brahme NN, Calderwood DA (2014) Integrin cytoplasmic tail interactions. Biochemistry 53: 810–820. 10.1021/bi401596q 24467163 PMC3985435

[4] Thomas GJ, Nystrom ML, Marshall JF (2006) Alphavbeta6 integrin in wound healing and cancer of the oral cavity. J Oral Pathol Med 35: 1–10. 16393247 10.1111/j.1600-0714.2005.00374.x

[5] Campbell ID, Humphries MJ (2011) Integrin structure, activation, and interactions. Cold Spring Harb Perspect Biol 3. 10.1101/cshperspect.a004994 PMC3039929 21421922

[6] Xu MY, Porte J, Knox AJ, Weinreb PH, Maher TM, Violette SM, et al (2009) Lysophosphatidic Acid Induces {alpha}v{beta}6 Integrin-Mediated TGF-{beta} Activation via the LPA2 Receptor and the Small G Protein G{alpha}q. Am J Pathol 174: 1264–1279. 10.2353/ajpath.2009.080160 19147812 PMC2671359

[7] Breuss JM, Gallo J, DeLisser HM, Klimanskaya IV, Folkesson HG, Pittet JF, et al (1995) Expression of the beta 6 integrin subunit in development, neoplasia and tissue repair suggests a role in epithelial remodeling. J Cell Sci 108 (Pt 6): 2241–2251. 7673344 10.1242/jcs.108.6.2241

[8] Bandyopadhyay A, Raghavan S (2009) Defining the role of integrin alphavbeta6 in cancer. Curr Drug Targets 10: 645–652. 19601768 10.2174/138945009788680374 PMC2888263

[9] Munger JS, Sheppard D (2011) Cross talk among TGF-beta signaling pathways, integrins, and the extracellular matrix. Cold Spring Harb Perspect Biol 3: a005017 10.1101/cshperspect.a005017 21900405 PMC3220354

[10] Juven-Gershon T, Kadonaga JT (2010) Regulation of gene expression via the core promoter and the basal transcriptional machinery. Dev Biol 339: 225–229. 10.1016/j.ydbio.2009.08.009 19682982 PMC2830304

[11] Bates RC, Bellovin DI, Brown C, Maynard E, Wu B, Kawakatsu H, et al (2005) Transcriptional activation of integrin beta6 during the epithelial-mesenchymal transition defines a novel prognostic indicator of aggressive colon carcinoma. J Clin Invest 115: 339–347. 15668738 10.1172/JCI23183 PMC544606

[12] Azare J, Leslie K, Al-Ahmadie H, Gerald W, Weinreb PH, Violette SM, et al (2007) Constitutively activated Stat3 induces tumorigenesis and enhances cell motility of prostate epithelial cells through integrin beta 6. Mol Cell Biol 27: 4444–4453. 17438134 10.1128/MCB.02404-06 PMC1900039

[13] Sullivan BP, Kassel KM, Manley S, Baker AK, Luyendyk JP (2011) Regulation of transforming growth factor-beta1-dependent integrin beta6 expression by p38 mitogen-activated protein kinase in bile duct epithelial cells. J Pharmacol Exp Ther 337: 471–478. 10.1124/jpet.110.177337 21303922 PMC3083106

[14] Abiko Y, Arai J, Mitamura J, Kaku T (1997) Alteration of proto-oncogenes during apoptosis in the oral squamous cell carcinoma cell line, SAS, induced by staurosporine. Cancer Lett 118: 101–107. 9310266 10.1016/s0304-3835(97)00234-6

[15] Li H, Fan H, Wang Z, Zheng J, Cao W (2013) Potentiation of scutellarin on human tongue carcinoma xenograft by low-intensity ultrasound. PLoS One 8: e59473 10.1371/journal.pone.0059473 23536878 PMC3607613

[16] Heckman KL, Pease LR (2007) Gene splicing and mutagenesis by PCR-driven overlap extension. Nat Protoc 2: 924–932. 17446874 10.1038/nprot.2007.132

[17] Breous E, Wenzel A, Loos U (2005) Promoter cloning and characterisation of the transcriptional regulation of the human thyrostimulin A2 subunit. Mol Cell Endocrinol 245: 169–180. 16376481 10.1016/j.mce.2005.11.009

[18] Deng X, Xu M, Yuan C, Yin L, Chen X, Zhou X, et al (2013) Transcriptional regulation of increased CCL2 expression in pulmonary fibrosis involves nuclear factor-kappaB and activator protein-1. Int J Biochem Cell Biol 45: 1366–1376. 10.1016/j.biocel.2013.04.003 23583295

[19] Duran EM, Shapshak P, Worley J, Minagar A, Ziegler F, Haliko S, et al (2005) Presenilin-1 detection in brain neurons and FOXP3 in peripheral blood mononuclear cells: normalizer gene selection for real time reverse transcriptase pcr using the deltadeltaCt method. Front Biosci 10: 2955–2965. 15970549 10.2741/1751

[20] Wang C, Lee J, Deng Y, Tao F, Zhang LH (2012) ARF-TSS: an alternative method for identification of transcription start site in bacteria. Biotechniques 0: 1–3. 10.2144/000113926 26307248

[21] Tora L, Timmers HT (2010) The TATA box regulates TATA-binding protein (TBP) dynamics in vivo. Trends Biochem Sci 35: 309–314. 10.1016/j.tibs.2010.01.007 20176488

[22] Lopez-Rodriguez C, Botella L, Corbi AL (1997) CCAAT-enhancer-binding proteins (C/EBP) regulate the tissue specific activity of the CD11c integrin gene promoter through functional interactions with Sp1 proteins. J Biol Chem 272: 29120–29126. 9360988 10.1074/jbc.272.46.29120

[23] Bansal M, Kumar A, Yella VR (2014) Role of DNA sequence based structural features of promoters in transcription initiation and gene expression. Curr Opin Struct Biol 25C: 77–85. 10.1016/j.sbi.2014.01.007 24503515

[24] Chatterjee S, Pal JK (2009) Role of 5′‐and 3′‐untranslated regions of mRNAs in human diseases. Biology of the Cell 101: 251–262. 10.1042/BC20080104 19275763

[25] Kadonaga JT (2012) Perspectives on the RNA polymerase II core promoter. Wiley Interdiscip Rev Dev Biol 1: 40–51. 10.1002/wdev.21 23801666 PMC3695423

[26] Starr DB, Hoopes BC, Hawley DK (1995) DNA bending is an important component of site-specific recognition by the TATA binding protein. J Mol Biol 250: 434–446. 7616566 10.1006/jmbi.1995.0388

[27] Zhao Y, Zhang J, Xia H, Zhang B, Jiang T, Wang J, et al (2012) Stat3 is involved in the motility, metastasis and prognosis in lingual squamous cell carcinoma. Cell Biochem Funct 30: 340–346. 10.1002/cbf.2810 22302289

[28] Yu H, Pardoll D, Jove R (2009) STATs in cancer inflammation and immunity: a leading role for STAT3. Nat Rev Cancer 9: 798–809. 10.1038/nrc2734 19851315 PMC4856025

[29] Nerlov C (2007) The C/EBP family of transcription factors: a paradigm for interaction between gene expression and proliferation control. Trends in cell biology 17: 318–324. 17658261 10.1016/j.tcb.2007.07.004

[30] Kumar M, Witt B, Knippschild U, Koch S, Meena JK, Heinlein C, et al (2013) CEBP factors regulate telomerase reverse transcriptase promoter activity in whey acidic protein‐T mice during mammary carcinogenesis. International Journal of Cancer 132: 2032–2043. 10.1002/ijc.27880 23023397

[31] Loomis KD, Zhu S, Yoon K, Johnson PF, Smart RC (2007) Genetic ablation of CCAAT/enhancer binding protein alpha in epidermis reveals its role in suppression of epithelial tumorigenesis. Cancer Res 67: 6768–6776. 17638888 10.1158/0008-5472.CAN-07-0139 PMC3773581

[32] Koschmieder S, Halmos B, Levantini E, Tenen DG (2009) Dysregulation of the C/EBPalpha differentiation pathway in human cancer. J Clin Oncol 27: 619–628. 10.1200/JCO.2008.17.9812 19075268 PMC2645860

[33] Zaragoza K, Begay V, Schuetz A, Heinemann U, Leutz A (2010) Repression of transcriptional activity of C/EBPalpha by E2F-dimerization partner complexes. Mol Cell Biol 30: 2293–2304. 10.1128/MCB.01619-09 20176812 PMC2863587

