# Supplementary material for: Cloning and Characterization of the Human Integrin β6 Gene Promoter
Source: PLoS One. 2015 Mar 27;10(3):e0121439. doi: 10.1371/journal.pone.0121439 (PMC4376883; doi:10.1371/journal.pone.0121439)

**Figure S1. Mutation analysis of AP1- and c-Myb-binding sites in the -289/-150 region of the ITGB6 promoter.** Luciferase activity expressed by TCA8113 and 293T cells after transfection with the potential AP1-binding site-directed mutant pGL2-B6-M-AP-1(A), the potential c-Myb-binding site-directed mutant pGL2-B6-M-cMyb(B), and wild-type construct pGL2-B6(-289/-150) 48 hr post transfection.


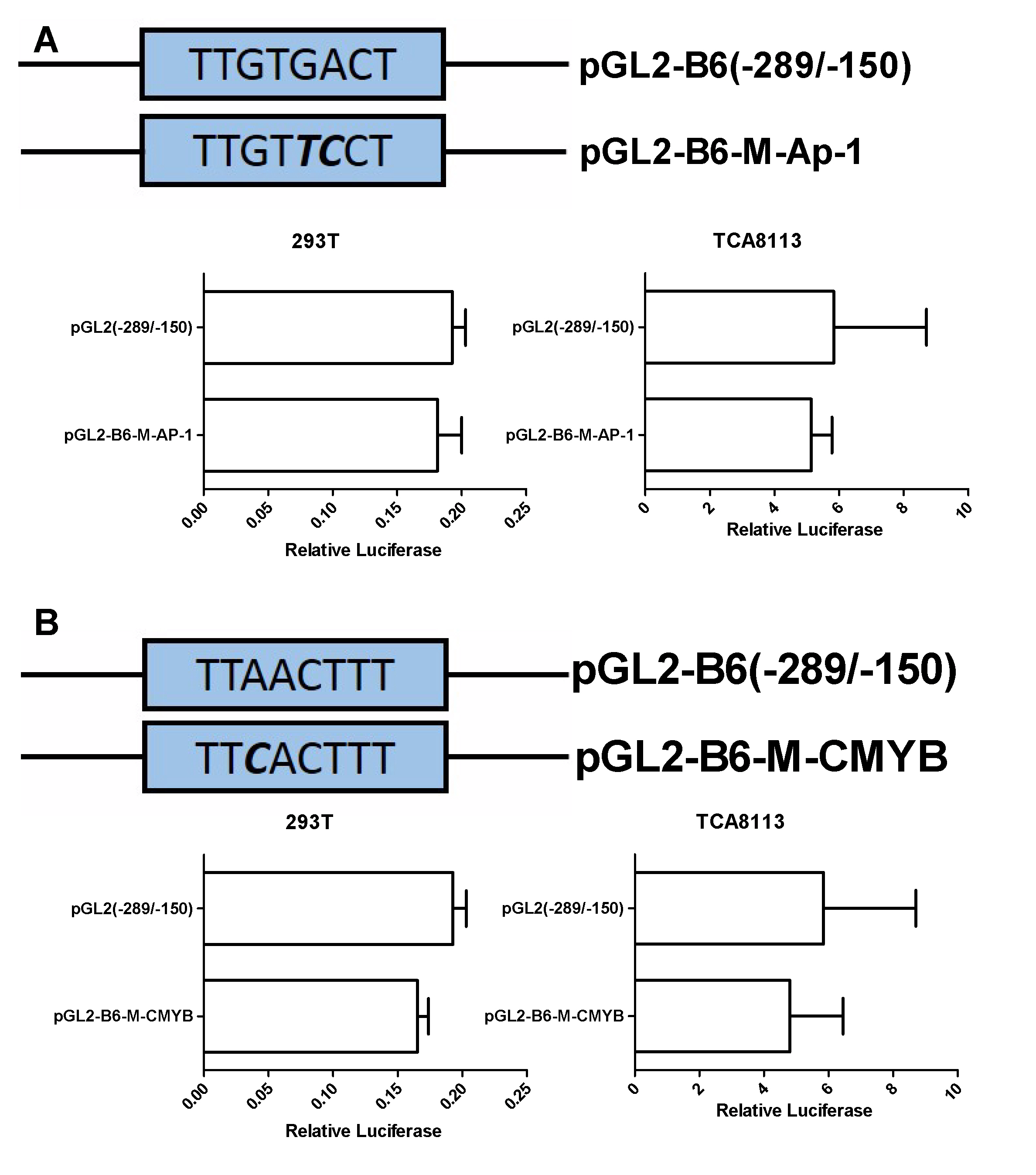

Supplement: S1 Fig — Luciferase activity expressed by TCA8113 and 293T cells after transfection with the potential AP1-binding site-directed mutant pGL2-B6-M-AP-1(A), the potential c-Myb-binding site-directed mutant pGL2-B6-M-cMyb(B), and wild-type construct pGL2-B6(−289/−150) 48 hr post transfection. (DOC) [file pone.0121439.s001.doc]
